# Targeting NF-κB pathway for treating ulcerative colitis: comprehensive regulatory characteristics of Chinese medicines

**DOI:** 10.1186/s13020-020-0296-z

**Published:** 2020-02-10

**Authors:** Peng-De Lu, Yong-Hua Zhao

**Affiliations:** 1 School of Pharmaceutical Sciences, Guangzhou University of Chinese Medicine, Guangzhou, China; 2 State Key Laboratory of Quality Research in Chinese Medicine, Institute of Chinese Medical Sciences, University of Macau, Taipa, 999078 Macao, Special Administrative Region of China

**Keywords:** Ulcerative colitis, Chinese medicines, NF-κB, Characteristics

## Abstract

Nuclear factor-kappa B (NF-κB) is a kind of multi-functional nuclear transcription factor involved in regulating gene transcription to influence pathological evolution of inflammatory and immune diseases. Numerous literature evidence that NF-κB pathway plays an essential role in pathogenic development of ulcerative colitis (UC). UC is a chronic non-specific inflammatory bowel disease, and until now, therapeutic agents for UC including aminosalicylates, corticosteroids and immune inhibitors still cannot exert satisfied effects on patients. In recent years, Chinese medicines suggest the advantages of alleviating symptoms and signs, decreasing side-effects and recurrence, whose one of mechanisms is related to regulation of NF-κB pathway. In this review, we categorize Chinese medicines according to their traditional therapeutic functions, and summarize the characteristics of Chinese medicines targeting NF-κB pathway in UC treatment. It indicates that 85 kinds of Chinese medicines’ compounds and formulae can directly act on NF-κBp65; while 58 Chinese medicines’ ingredients and formulae indirectly suppress NF-κBp65 by regulation of its upstream or other related pathways. Moreover, by the analysis of Chinese medicines’ category based on their traditional functions, we conclude the category of dampness-drying and detoxificating medicine targeting NF-κB pathway accounts for primary status for amelioration of UC. Simultaneously, this review also contributes to the choices of Chinese medicine category and provides curative potential of Chinese medicines for clinical UC treatment.

## Background

In recent years, the incidence and prevalence of global ulcerative colitis (UC) have shown a rapidly increasing trend. It suggests that the highest prevalence rate of UC (505/100,000 in Norway) exists in western Europe, and the prevalence rate of UC in Korea increases from 0.34/100,000 in 1986–1990 to 3.08/100,000 in 2001–2005 [1, 2]. Statistical data show that the prevalence rates of Heilongjiang, Xian, Guangzhou, Zhongshan, Hong Kong and Taiwan are 1.77/100,000, 0.42/100,000, 3.44/100,000, 3.14/100,000, 3.06/100,000 and 4.59/100,000, respectively [3–5]. Moreover, compared with UC in western countries, the disease in China is found to have some differences in clinical characteristics [6].

Typical symptoms of UC include diarrhea, mucus pus, bloody stools and abdominal pain, and its time of onset is more than 4 to 6 weeks, simultaneously, extraintestinal manifestations involve skin mucosa, joints, eyes, lungs and nervous system [7–15]. Additionally, diarrhea, which does not exceed 6 weeks of disease process, needs to be distinguished from infectious enteritis [16]. Presently, western medicine generally thinks that there are several pathological factors related to UC, including genetic factor, infectious factor, intestinal microbiota imbalance, immune response, dietary factor and so on. Study evidences that less than 20% of susceptibility genes are associated with UC and 28 functional chromosomal loci have been reported [17]. A 10-year study on cytomegalovirus colitis shows that it has been a poor prognostic indicator among patients with UC onset [18]. Probiotics have been demonstrated to be able to protect intestinal mucosal barrier and reduce intestinal inflammation, and evidences indicate that a protective role of bifidobacteria in experimental induced colitis is related to regulation of an innate immune response of the host, and immune homeostasis contributes to the reduction of inflammatory bowel disease (IBD) onset [19, 20]. With the improvement of people’s living standards, the dietary structure begins to change. The intakes of meat and egg-milk products gradually increase and dietary fiber-based foods decreases. This high-protein and low-vegetable fiber diet has become one of the risk factors for enhancing prevalence of UC [21]. Moreover, excessive intakes of high-calorie and heterologous proteins may stimulate the body’s immune response, leading to aggravation or recurrence of UC, while diet strategy of reducing unsaturated fatty acids in food is effective on UC treatment [22].

Current therapeutic drugs for UC include 5-aminosalicylic acid (5-ASA), glucocorticoids, immunomodulators, biological agents and traditional Chinese medicines. The evidence-based consensus released by European Cron’s and Colitis Organisation (ECCO) recommends 5-ASA as the primary drug for the treatment of mild to moderate UC and maintaining drug during remission [23]. An analysis on mild to moderate UC and remission of UC shows that 50.5% patients with local treatment and 41.3% patients with oral treatment achieve clinical amelioration, but the incidence of adverse reactions in local and oral treatments was 22/105 (21.0%) and 36/109 (33.0%), respectively [24]. Glucocorticoids are prone to hormone dependent and resistant. In the treatment using budesonide foam enema for mild to moderate UC, the rate of side effects was 45.9% [25]. Therefore, glucocorticoids are not suitable for long-term application for maintaining treatment. Immunosuppressive agents have more significant adverse reactions such as nausea, vomiting, abdominal pain, severe diarrhea, myelosuppression, hepatotoxicity and pancreatitis, and in the treatment of biological agents, a large number of patients do not get mucosal healing and the mucosal healing also does not mean that the symptoms disappear [26, 27]. Although the above drugs have achieved a certain extent effects on clinical remission of UC and promoting mucosal healing, it still cannot meet the requirements of UC treatment. Clinically, Chinese medicines not only obviously improve symptoms and accelerate ulcer healing, but also alleviate adverse reactions of western medicine and enhance patients’ quality of life. Therefore, Chinese medicines have become a hotspot in UC treatment in recent years [28–32].

Data show that 20.1% of UC patients in China accept the treatment of Chinese herbs, and 59.1% are administered with combined Chinese with western medicines [33]. Moreover, Chinese medicines are reported to be satisfied efficacious in the treatment for mild to moderate UC [34]. It shows that combination treatment of Chinese medicine formula and mesalazine notably improves the intestinal symptoms, mucosal condition, and extraintestinal characterizations compared to mesalazine alone in UC patients after 8 weeks [35]. Another study indicates that the therapeutic effect of curcumin combined with mesalamine is superior to that of mesalamine in patients with mild-to-moderate UC, and no apparent side effects emerge [36].

Although precise mechanisms are still not clear at present, numerous literature suggest that NF-κB pathway plays a central role in regulating the release of cytokines in patients with UC and participates in the inflammation and immune response in the intestinal tract of UC [37–39]. The imbalance between excessive secretion of pro-inflammatory cytokines and relative insufficient secretion of anti-inflammatory cytokines is linked to the development of non-specific inflammatory responses in the intestine [40]. It indicates that NF-κBp65 highly expresses in intestinal mucosal epithelium, crypt epithelial cells and lamina propria monocytes of patients with UC, and the expression of NF-κB in the nucleus is significantly higher than that in cytoplasm [41]. NF-kappaB p65 antisense oligonucleotides blocks NF-κB pathway and down-regulates NF-κB-dependent IL-1beta and IL-8 mRNA expressions, which attenuates the productions of pro-inflammatory cytokines in lamina propria mononuclear cells from patients with UC [42]. In this review, based on the therapeutic superiorities of Chinese medicines and the important role of NF-κB pathway in UC pathogenesis, we summarize Chinese medicines and their active compounds as well as Chinese medicine formulae targeting NF-κB signaling pathway, and further analyze the characteristics and advantages of Chinese medicines during the process of UC treatment.

## Traditional Chinese medicine’s pathogenesis of UC and therapeutic strategy

According to the etiology and pathogenesis theory of traditional Chinese medicine, the pathogens of dampness-heat and dampness-cold account for predominant status in the onset of UC. Subsequently, qi stagnation and blood stasis present in intestine, which exacerbates the symptoms of UC, e.g., abdominal pain, diarrhea, mucus pus and bloody stools. Additionally, emotion disorder and unhealthy diet lead to the disharmonization of liver and spleen functions, triggering cold and heat in complexity resulting in the change of stomach-intestine environment. Consequently, the pathological development injures spleen qi and kidney yang, causing the deficiency of spleen and kidney functions. Patients with chronic UC are prone to attract complications by exopathogenic factors, because it always exists deficiency in spleen qi and kidney yang. Therefore, UC is commonly divided into “syndrome of dampness-heat”, “syndrome of deficiency-cold of spleen and stomach”, “syndrome of deficiency of spleen and kidney Yang” and “syndrome of cold and heat in complexity”, “syndrome of qi stagnation and blood stasis” and so on [43]. In the treatment of UC, clearing heat, drying dampness, detoxifying, and activating qi and blood, harmonization of cold and warm, warming yang, tonifying qi are regular therapeutic methods.

## Chinese medicines and their active compounds targeting NF-κB pathway

At present, regulation of NF-κB pathway has become a key strategy in the treatment of UC. According to traditional Chinese medicinal functions, we categorize Chinese medicines and their active compounds targeting NF-κB pathway into seven types.

### Heat-clearing and dampness-drying medicine

Casticin, a compound extracted from *Vitex Fructus*, showing anti-inflammatory and antitumor effects. Research suggests that casticin (10 mmol/L) treatment alleviates body weight loss, colon length shortening and pathological damage in the colon of dextran sulfate sodium (DSS)-treated mice, as well as inhibits the productions of pro-inflammatory chemocytokines through down-regulating AKT/NF-κB pathway in macrophages [44].

Baicalin, wogonin and wogonoside are abundant in roots of *Scutellaria baicalensis* Georgi. It is reported that baicalin (50, 100 or 150 mg/kg) treatment significantly reduces interleukin (IL)-33 and NF-κBp65 levels, and increases IκBα levels against the severity of UC in DSS-induced mice [45]. Moreover, treatment with baicalin (20 and 40 μmol/L) obviously up-regulates expression of IL-4 and IL-10, increases ratio of p-STAT6/STAT6, but decreases ratios of p-STAT4/STAT4 and p-NF-κB/NF-κB compared to the treatment of no baicalin in UC patients [46]. In addition, evidence indicates that wogonin (25 mg/mL) treatment obviously attenuates the inflammatory response of toll like receptor (TLR4)-myeloid differentiation factor (MyD) 88-mediated NF-κB pathway in lipopolysaccharide (LPS)-induced intestinal inflammation of Caco-2 cells in vitro, suggesting protective function on intestinal mucosal barrier [47]. As for wogonoside (12.5, 25 or 50 mg/kg), its application inhibits the activation of NF-κB-induced NLRP3 inflammasomes in DSS-induced colitis and colitis-associated tumorigenesis in mice [48, 49].

*Coptis chinensis* Franch. has the highest use frequency for UC treatment. As a main ingredient of *C. chinensis* Franch., berberine (100 mg/kg) treatment inhibits the activations of NF-κB and mitogen-activated protein kinase (MAPK), which contributes to down-regulation of tumor necrosis factor (TNF), interferon gamma (IFN-γ) and IL-17 expressions in colonic macrophages and epithelial cells from DSS-induced mice, showing the reductions of crypt injury and severe inflammatory damage [50]. In addition, berberine (100 mg/kg or 100 μM) treatment improves intestinal barrier function through suppression of the phosphorylated colonic signal transducer and activator of transcription (STAT) 3 and myosin light chain (MLC) kinase-MLC signaling pathway, as well as inhibition of IFN-γ and TNF-α expressions and macrophage infiltration into the intestinal mucosa in DSS-colitis mice [51, 52].

As a derivative of coptisine, (±)-8-ADC (75, 150, and 300 mg/kg) treatment activates the transcriptional activity of X-box binding protein 1 and decreases NF-κB expression, subsequently reduces secretion of myeloperoxidase, TNF-α, IL-6 and IL-1β in the colon of DSS-induced colitis mouse. Over all, it significantly inhibits the development of colitis and improves the pathology associated with acute colitis induced by DSS [53].

Berberine hydrochloride is one of the effective compounds among *Rhizoma Coptidis*, *Cortex Phellodendri*, and other plants. Study has demonstrated that berberine hydrochloride (10, 30 and 50 mg/kg) treatment is able to suppress the expressions of phosphorylation of STAT3 and phosphorylation of NF-κBp65 which are induced by DSS in colonic tissues. Moreover, berberine hydrochloride treatment can effectively inhibit the activation of IL-6/STAT3/NF-κB in an experimental UC model in vivo, suggesting gut protective effects [54, 55].

As a constituent of *Picrorhiza kurroathe*, apocynin (control diet with 2% apocynin) treatment significantly reduces the activation of NF-κBp65 as well as STAT3 in DSS-treated colon tissues and the colitis induced by DSS in mice [56].

Matrine and oxymatrine are isolated from *Sophora flavescens* Ait. or *Sophora subprostrata* Chun et T.Chen. Studies suggest that in trinitrobenzene sulfonic acid (TNBS)-induced UC rats, matrine (180 mg/kg) treatment decreases the lesion area of inflammatory cell infiltration, edema and fibrosis and IL-1β, TNF-α, IL-6, IL-8 levels in colonic tissues. Kushenin injection (63 mg/kg) lows the overexpression of colonic mucosa proteins NOD2 and NF-κBp65 and decreases IL-6 secretion, which contributes to the attenuation of UC [57, 58]. Oxymatrine (63 mg/kg) is found to reduce intestinal mucosa injury via up-regulation of the β2-adrenoceptor and β-arrestin2 expressions and down-regulation of NF-κBp65 expression in colonic mucosa and spleen lymphocytes from TNBS-induced UC rats [59].

*Ampelopsis grossedentata* (AMP), which contains a large amount of flavonoid active compounds, has the traditional function of clearing heat and detoxification. AMP (400 mg/kg) exerts protective effects on DSS-induced UC via suppressing the IL-1 receptor associated kinase (IRAK1)/TNF receptor-associated factor 6 (TRAF6)/NF-κB-mediated inflammatory signaling pathway [60].

SM934 (3, 10 mg/kg) is a water-soluble artemisinin analogue that shows significant attenuation of DSS-induced colonic inflammatory responses by suppressing the effects of macrophages and neutrophils and inhibiting the NF-κB signaling pathway [61]. Additionally, artesunate (10 30, and 50 mg/kg), a semi‑synthetic derivative of artemisinin, exerts anti‑inflammatory effects via regulating the TLR4‑NF‑κB signaling pathway in DSS-induced UC of rats [62].

Tetrandrine is a bisbenzylisoquinoline alkaloid extracted from the roots of *Stephania tetrandra*, and oral administration of tetrandrine (65 mg/kg) has distinct therapeutic effects on DSS-induced UC of mice, including inhibiting weight loss and improving diarrhea and blood stool. Its mechanism may be related to inhibition of the binding activity of NF-κB to DNA and down-regulation of the levels of TNF-α and IL-1β in colon tissue [63].

Evidence shows that *Polygonum cuspidatum* Siebold & Zucc. (PCS) treatment can prevent DSS-caused weight loss and colon length reduction in UC mice, as well as increase the serum levels of IL-10 and reduce the levels of IL-1β, IL-6 and TNF-α. Moreover, whatever PCSE (100, 200 and 400 mg/kg) or the combination of its three extractions: polydatin, resveratrol and emodin exhibit higher inhibitory activities for cytokines and NF-κB signaling related molecules than any one of the three compounds with same concentration treatment [64].

### Interior-warming and drying dampness medicine

Aesculin is a main active compound of *Aesculus chinensis* Bunge with strong anti-inflammatory action. Aesculin (1, 5 mg/kg) treatment can attenuate the activity of NF-κB signaling and promote the nuclear localization of peroxisome proliferator-activated receptor gamma (PPAR-γ) in both rectal tissues from DSS-induced mice and LPS-stimulated macrophages, suggesting the protection of aesculin against UC might attribute to its regulation on the PPAR-γ and NF-κB pathway [65].

Papaya [*Chaenomeles speciosa* (Sweet) Nakai] is either Chinese medicine or food. The main active compound of papaya, total triterpenoids of *C. speciosa* (50, 100 mg/kg) treatment can significantly reduce the levels of pro-inflammatory factors such as TNF-α, IL-1β, IL-6, IFN-γ, the ratios of p-IKKβ/IKKβ and p-IκBα/IκBα, and nucleus NF-κBp65 in the colon tissue, while increase the levels of anti-inflammatory factors IL-4 and IL-10 in the blood, as well as the expressions of PPARγ in the nucleus, SIRT1 in tissue and NF-κBp65 in cytoplasm, which contributes to the improvements of the general symptoms and pathological evolution of DSS-induced UC model mice [66].

Cardamonin is found in roots of *Alpinia* katsumadai Hayata. It (1–50 mg/kg) has been found to block nuclear translocation of NF-κBp65 in a mouse model of endotoxin shock, contributing to the attenuation of TNF-α, IL-6 and IL-1β secretions in LPS-induced mouse blood serum [67], and inhibit NF-κB DNA-binding in LPS-stimulated macrophage cells [68]. In acetic acid-induced colitis model, cardamonin (10 or 30 mg/kg) treatment ameliorates the disease activity index (DAI) and macroscopic damage index scores, and reduces the levels of NF-κB and TNF-α as well as oxidative stress and apoptosis, evidencing it has a protective effect against acetic acid-induced colitis [69].

3, 4-Oxo-isopropylidene-shikimic acid (ISA) is a derivative of shikimic acid (a monomeric compound extracted from *Illicium verum* Hook.f.). In TNBS-induced colitis of rats, ISA (50, 100, 200 mg/kg) treatment not only exerts anti-oxidative and inhibits arachidonic acid actions, but also increases IκBα protein expression and decreases NF-κBp65 protein expression in the colonic mucosa, showing significant therapeutic effects on experimental colitis in rats [70].

The main compounds of the chloroform extract of Wu Zhu Yu (WZY/*Euodia rutaecarpa* (Juss.) Benth.) are evodiamine and rutaecarpine. It shows that evodiamine (80 mg/kg) treatment notably improves body weight loss, DAI, colon length shortening and colonic pathological damage in DSS-induced UC mice, and decreases phosphorylation levels of NF-κBp65 and IκB and inhibits NLRP3 inflammasome activation in colonic tissues [71].

Flower bud and fruit of Clove (*Eugenia caryophyllata* Thumb.) are as medicinal application, and their main active compound is iridoid glycosides. Study evidences that iridoid glycosides (80, 160 and 240 mg/kg) have distinct therapeutic effects on TNBS-induced UC mice, embodying reduction of the protein and mRNA expressions of NF-κBp65, TNFα and IL-6 in the colon tissues [72]. Additionally, symptoms and weight loss present significant amelioration in mice induced by DSS under the treatment with syringic acid (25 mg/kg) extracted from clove. What’s more, its application decreases the expressions of NF-κBp65 and IκB in LPS-induced RAW 264.7 cells, thus reducing the activation and nuclear accumulation of p-STAT-3Y705, which contributes to inhibitions of IκB degradation and the nuclear translocation of NF-κBp65 in colonic tissue [73].

In DSS-induced experimental mice colitis, it suggests that *Zanthoxylum bungeanum* pericarp extract (ZBE) (0.5, 1, 2 g/kg) treatment alleviates body weight loss, colon length shortening and colonic pathological damage, whose mechanisms are related to inhibiting NF-κBp65 and IκBα phosphorylation levels in a dose dependent manner and LPS-induced J774.1 cells. In addition, the suppression of NF-κB and MAPK pathways is partly attributed to the inhibition of their upstream TLR4 [74].

Research has found that galangin (20 and 40 mg/kg) isolated from *Alpinia officinarum* treatment either reduces the levels of nitrites, IL-6, and TNF-α in LPS-stimulated RAW 264.7 cells in a concentration-dependent manner, or decreases the levels of TNF-α and IL-6 in colonic tissue and serum, as well as increases levels of anti-inflammatory cytokine (IL-10). Moreover, its application also suppresses protein expressions of p-NF-κB and p-Ikk-βα in colon tissues against inflammatory injury in mice [75].

### Detoxificating and purgative medicine

Parthenolide is the main active compound of the genus *Tanacetum parthenium*. Studies reveal that parthenolide is used for the remedy of inflammatory diseases such as arthritis, asthma, and so on, and has been a potent inhibitor of NF-κB activation in cultured cells and experimental models. In DSS-induced mice UC, parthenolide (5, 10 and 15 mg/kg) is capable of inhibiting the secretions of TNF-ɑ and IL-1β in colon tissue via blocking the phosphorylation and degradation of IκBα and suppressing the phosphorylation of NF-κBp65, suggesting beneficial effects in experimental colitis [76]. Moreover, cotreatment with parthenolide and balsalazide (20 ng/mL) shows a significant inhibition in the TNF-α-induced phosphorylation of IkB-α and also blocks TNF-α-induced activation of p65 in HCT116 cells, suggesting that parthenolide enhances the effect of balsalazide on inhibition of NF-κB activation [77].

Research shows that arctigenin (25, 50 mg/kg) derived from *Arctium lappa* L. treatment significantly suppresses the phosphorylated MAPKs and the activation of NF-κB by the inhibitions of phosphorylated IκBα and p65, p65 translocation and DNA binding activity. Simultaneously, its application markedly recovers the loss of intestinal epithelial cells, and decreases the infiltrations of neutrophils and macrophages and the secretion of inflammatory cytokines in DSS-induced UC mice [78].

Andrographolide is a natural antibiotic drug mainly existed in *Andrographis paniculata* (Burm. f.) Nees. It is reported that the andrographolide derivative AL-1 (andrographolide-lipoic acid conjugate) (5, 15 and 45 mg/kg) treatment can attenuate the expression levels of p-IκBα, p-p65 and cyclooxygenase-2 (COX2), and increase the expression of PPAR-γ, which contributes to reduction of colonic damage in TNBS-induced mice colitis [79]. In addition, another andrographolide (200 mg/kg) derivative CX-10, a semi-chemical synthesized by andrographolide, reduces the expressions of IL-6 and TNF-α in colon tissue, as well as the expressions of NF-κBp65 and p-IκBα. Meanwhile its application increases the expression of IκBα and suppresses the phosphorylated p38MAPK, extracellular regulated protein kinases (ERK) and c-Jun N-terminal kinase (JNK). The significant effects ameliorate the colitis symptoms in DSS-induced UC mice [80].

Brusatol (BR) is one of the main active compounds of *Brucea javanica* (Linn.) Merr. It is reported that BR (0.25, 0.5, 1 mg/kg) treatment can inhibit the levels of pro-inflammatory cytokines and prostaglandin E2 (PGE2), and promote the production of immunoregulatory mediators like IL-4 and IL-10. Moreover, treatment with aqueous solution of BR significantly attenuates the expressions of TLR4, MyD88 and NF-κBp65 in colon tissue. It suggests that BR treatment is able to alleviate a certain degree of symptoms in mice with colonic inflammation by regulation of anti-inflammatory status [81]. Additionally, the ethyl acetate extract of the ripe fruit of *B. javanica*, brucea javanica oil (BJO) (0.5 1 and 2 g/kg) treatment reduces the body weight loss, restores the colon length and decreases the levels of TNF-α, IL-1β, IL-6, IL-8, IL-17 and IFN-γ via the suppression of NF-κB pathway in DSS-induced mice [82].

Total flavonoids of *Hedyotis diffusa* Willd (50, 100, 200 μg/mL) is reported to exert its anti-inflammatory activity via reducing pro-inflammatory nitric oxide, TNF-α, IL-6 and IL-1β levels in LPS-stimulated RAW 264.7 cells through the suppressions of IκB phosphorylation resulting in inactivation of NF-κB pathway and the phosphorylation of MAPK signaling pathway [83]. Moreover, administration of *Oldenlandia diffusa* (OD) (1 g/kg) attenuates symptoms induced by DSS in mice, including the ameliorations of weight loss and colon length. Moreover, its application suppresses levels of IL-6, IL-1β and expression of COX2 in DSS-treated colon tissues, which is related to inactivation of NF-κBp65 in colon tissues [84].

Qing Dai (QD) is an extraction from leaves or stems of *Strobilanthes cusia* (Nees) Kuntze, *Polygonum tinctorium* Ait. and *Isatis indigotica* Fortune. Study has demonstrated that QD (120, 300 or 600 mg/kg) treatment distinctly improves the pathological degree of UC via increasing the mRNA expressions of IL-10 and IL-22 in lymphocytes [85]. Additionally, QD (0.77, 1.54 and 3.08 g/kg) treatment reduces DSS-induced macrophage infiltration and TNF-α, IL-1β, IL-6 expressions in colonic tissues, and decreases TNF-α, IL-6 and COX-2 expressions via blocking LPS-induced IκBα degradation and p65 nuclear translocation in RAW264.7 cells [86].

Indirubin is a compound extracted from Danggui Longhui Wan. It shows that indirubin (10 mg/kg) treatment obviously alleviates the symptoms and signs of DSS-induced UC in mice, and decreases inflammatory cell infiltration in the colon mucosa. Simultaneously, its application can regulate inflammatory cytokines, embodying the down-regulation of TNF-α, IFN-γ and IL-2 levels and up-regulation of IL-4 and IL-10 levels in colon tissues, which is related to inhibition of DSS-induced activation of NF-κB signaling [87].

Dandelion (*Taraxacum mongolicum* Hand.-Mazz.) contains various active compounds such as dandelion sterol, choline, inulin and pectin. Its pharmacological activities include anti-inflammatory, immune regulation, free radical scavenging, and anti-tumor. Evidence shows that aqueous dandelion root extract (20 mg/kg) has anti-inflammatory effects on human colonic epithelial cells by increasing DSS-reduced cell viability and attenuating DSS-induced apoptosis, reactive oxygen species (ROS), and NF-κB signal activation [88].

*Portulaca oleracea* L., (POL) is one of commonly used medicinal-food herbs. Experimental results demonstrate that the ethanol extract from POL (100, 200 and 400 mg/kg) treatment increases the colon length, decreases body weight loss and reduces the mRNA expressions of TNF-α, IL-1β and IL-6 and the protein expressions of TNF-α and NF-kBp65 in DSS-induced C57BL/6 mice UC model [89]. Additionally, therapeutic effects of portulaca extract (100 mg/kg) is similar to that of mesalazine, which presents reducing the expression levels of these cytokines including TNF-α, IL-6, and IL-1β and the level of DSS-induced NF-κB phosphorylation [90].

Rhubarb-type anthraquinones are from *Rheum palmatum* L., *Rheum tanguticum* Maxim. ex Balf., or the dried roots and rhizomes of *Rheum officinale* Baill., including rhein, emodin, chrysophanol, and aloe emodin. Studies have shown that rhein (1, 5 and 20 μM) treatment reduces nitric oxide (NO) production by suppressing the protein expressions of inducible nitric oxide synthase (iNOS) and COX-2, and its anti-inflammatory action is partially associated with reduction of the phosphorylation levels of NF-κBp65 and the suppression of NLRP3 expression in RAW264.7 macrophages [91]. It is reported that emodin (5, 10 and 20 mg/kg) treatment alleviates the symptoms and decreases the level of anti-flagellin in serum and suppresses the expressions of TLR5 and NF-κBp65 in colon of DSS-induced UC mice. Moreover, in vitro, emodin treatment inhibits the nuclear translocation of NF-κBp65 and decreases the release of IL-8 in flagellin-stimulated HT-29 cells via down-regulating the expressions of TLR5 and MyD88, up-regulating the expression of IκB [92]. Chrysophanol (5 mg/kg) treatment decreases the activation of NF-κBp65 and caspase-1 in DSS-treated colon tissue and LPS-stimulated murine peritoneal macrophages, which contributes to attenuation of overall clinical scores and various pathological markers of colitis via against inflammatory injury [93].

Gallic acid (GA) is mainly isolated from rhizomes, dried leaves, and fruits of plants such as *R. palmatum* L., *Eucalyptus robusta* Sm. Research indicates that GA (10 mg/kg) treatment can attenuate the activation and nuclear accumulation of p-STAT3 (Y705), prevent the degradation of the inhibitory protein IκB, and inhibit the nuclear translocation of NF-κBp65 in colonic mucosa of DSS-exposed mice model against inflammatory response [94].

Allicin is a sulfur-containing compound naturally found in the bulbs of the lily plant *Allium sativum* L. It is reported that allicin (10 mg/kg) exerts anti-inflammatory effects through reduction of mRNA levels of TNF-α, IL-1β, IL-6 and IL-17, which is related to inhibiting activation and nuclear accumulation of STAT3 and preventing the inhibition of protein IκB degradation and inducing nuclear translocation of NF-κBp65 in colonic mucosa [95].

Study has found that treatment with *Cassia obtusifolia* (CO) (1 g/kg) ameliorates signs including body weight loss and increased DAI, shortened colon length in DSS-induced colitis mice, as well as suppresses the levels of IL-6 and expression of COX-2. Its therapeutic mechanism is associated with reduction of the activation of NF-κBp65 in colon tissues [96].

Bergenin isolated from the herb of *Saxifraga stolonifera* Curt alleviates disease symptoms of mice with DSS-induced colitis. Its mechanisms embody that bergenin (20, 50 mg/kg) treatment activates PPARγ, leading to increased expression of SIRT1. Subsequently, SIRT1 reduces the acetylation of NF-κBp65, up-regulates the association of NF-κBp65 and IκBα. Consequently, the nuclear translocation of NF-κBp65 is prevented resulting in reduced expressions of TNF-α and IL-6 against inflammatory injury in DSS-induced mice [97].

*Fagopyrum cymosum* (Trev.) Meisn (Fag) (0.57, 1.14 and 2.28 g/kg) from Polygonaceae family is reported to inhibit the production of proinflammatory cytokines via inhibiting NF-κBp65 nuclear translocation and IκB phosphorylation in TNBS-induced colitis model of rats. Furthermore, the clinical study results reveal that Fag has fewer side effects and serves as a better anti-inflammatory potential drug for UC compared with salicylazosulfapyridine [98].

Chlorogenic acid from Chinese medicine honeysuckle, hawthorn, eucommia and chrysanthemum, has the ability to improve the expression of ERK1/2, p-ERK, p38, p-p38, JNK, and pJNK proteins in MAPK/ERK/JNK pathway in DSS-induced UC mice. Moreover, study has found that chlorogenic acid (30, 60 and 120 mg/kg) treatment can reduce or inhibit DSS-induced colonic mucosal damage, inflammation, oxidative stress, apoptosis and the phosphorylation level of IκB and NF-κBp65 protein in UC mice, thus inhibiting the activity of NF-κB [99].

### Blood-activating medicine

Paeonol is the main active compound isolated from *Moutan cortex*. It is reported that paeonol enema (100 μg/mL) treatment improves the symptoms and pathology including body weight, colon length and histological score, and reduces TNF-α-induced NF-κB transactivation and IFNγ-induced STAT1 transactivation in colon cancer-derived CW-2 cells and T cell leukemia-derived jurkat cells, suggesting paeonol enema may be useful for the treatment of colitis [100].

As main bioactive compound extracted from *Sargentodoxa cuneata*, Liriodendrin (100 mg/kg) treatment distinctly improves DAI, colon length and histological damage in colon and suppresses the levels of TNF-a, IL-1β and IL-6 and the activation of Akt and NF-κB pathways, and up-regulates the expression of ERβ in the colon of DSS-induced mouse colitis model. Simultaneously in vitro, liriodendrin treatment down-regulates production of pro-inflammatory cytokines and suppresses NF-κB signaling pathways in LPS-induced RAW 264.7 macrophages [101].

Shikonin is a purple flaky crystal or crystalline powder. It is the main active compound in the roots of *Lithospermum erythrorhizon* Sieb. et Zucc. Its pharmacological effects include anti-cancer, anti-inflammatory and antibacterial. Shikonin (6.25, 12.5 and 25 mg/kg) shows obvious therapeutic actions on DSS-induced acute colitis in Balb/C mice. Its application notably reduces the amount of CD4+ lymphocytes and macrophages in the colon tissue of mice, and the release of pro-inflammatory factors such as TNF-α, IL-1β, and IL-6 production. In addition, shikonin treatment also reduces the expressions of COX-2 and NF-κBp65, and phosphorylation of STAT-3 in IBD model [102].

Tetramethylpyrazine (TMP), also known as ligustrazine, is the main active compound purified from *Ligustium wollichii franchat*. It is found that TMP (80 mg/kg) treatment attenuates the damage caused by intrarectal instillation of oxazolone (OXZ) and substantially reduces the rise in TNF-α and NF-κBp65 expressions, and increases PPAR-γ production in colitis mice [103].

Crocetin is a constituent of saffron (*Crocus sativus*). Evidence indicates that crocetin (50 mg/kg) treatment improves diarrhea and the disruption of colonic architecture in TNBS-induced mice colitis model, and inhibits IL-12 production through the down-regulation of NF-κB-mediated activation and enhances IL-4 in CD4+ T cells, showing useful supplement therapy for UC [104].

Epicatechin is widely found in plants such as *Acacia catechu* (L.f.) Willci. Epicatechin. Study indicates that the doses of 100, 200 or 300 mg/kg can alleviate the DAI and colon macroscopic damage index scores, reduce body weight loss, and significantly relieve colon contracture and crypt damage in DSS-induced acute UC mice model, and reduce TNF-α, IL-6 and inhibit NF-κB pathway in vivo and in vitro [105]. Another similar component, (2)-epigallocatechin-3-gallate (EGCG), has inhibited effect on NF-κB nuclear translocation in IEC-6 cells of UC patients by the inactivation of IκB kinase [106].

Curcumin is a natural hydrophobic polyphenol, a compound mainly extracted from the rhizome of the ginger family *C. longa* L. [107], and exerts multiple pharmacological effects on IBD [108, 109]. Curcumin (100 mg/kg or 15, 30, 60 mg/kg) treatment not only down-regulates expression of pro-inflammatory cytokines, such as IL-1, IL-6, IL-8, and TNF-α by regulating NF-κB/IkB pathway, reduces inflammatory cell infiltration in several experimental models of UC, but also suggests clinical remission in active mild-to-moderate UC patients and reduces clinical relapse in quiescent UC patients [110–112].

### Qi-regulating medicine

Norisoboldine (NOR), the main active compound of *Radix Linderae*, indicates that the doses of 20, 40 mg/kg markedly reduce the symptoms of colitis and inhibit phosphorylations of ERK and p38MAPK, and phosphorylation, nuclear translocation and DNA-binding activity of NF-κBp65 in colons of UC mice, which might offer a reasonable explanation for inhibition of pro-inflammatory cytokines by NOR [113].

Research reveals that *Citrus aurantium* L. and its flavonoids (Naringenin, nobiletin, and hesperetin) (125, 250 and 500 mg/kg) suggests anti-inflammatory effects on TNBS-induced IBD and LPS-induced RAW cells, which is related to the inhibition of TNF-α-induced NF-κB pathway [114].

### Tonifying medicine

Study shows that inhibitory effects of *Eclipta Prostrata* (EP) (500 mg/kg) on DSS-induced UC occurrence by improving body weight loss, shortened colon length and DAI, and reducing the phosphorylation of IκB and the translocation of the NF-κB. Additionally, a HPLC analysis shows that wedelolactone, contained in water extract of EP, is an inhibitor of NF-κB transcription [115].

Astragalus polysaccharide (APS) and astragaloside IV (ASI) are extracts of *Astragalus membranaceus*. Study reveals that APS (200 mg/kg) treatment increases weight and colon length and reduces NF-κB DNA phosphorylation activity and down-regulates TNF-α, IL-1β, IL-6, IL-17 expressions associated with improvement in DSS-induced mice colitis [116]. In addition, ASI (200 mg/kg) treatment reverses the increase of TNF‑α, IL-β and IL‑6 inflammatory cytokine via the inhibition of the NF‑κB pathway in LPS-stimulated CCD-18Co cells. In DSS-induced UC mice, except for the inhibition of pro‑inflammatory cytokines, ASI treatment also decreases the level of p‑p65 and p‑IκB proteins, suggesting effective amelioration on experimental UC in vitro and in vivo [117].

Licochalcone A (Lico A) is a characteristic chalcone existing in licorice root, which is widely used in Chinese medicine formulae. The results show that Lico A (20, 40 and 80 mg/kg) treatment reduces the colon length, histological damage scores and MPO activity, and decreases the oxidative stress and pro-inflammatory cytokines, down-regulates NF-κB pathway and up-regulates Nrf2 pathway in DSS-induced UC [118]. Furthermore, as a substance extracted and purified from *Glycyrrhiza uralensis* Fisch, diammonium glycyrrhizinate (40 mg/kg) treatment is found to reduce inflammatory injury in a rat model of UC via suppressions of NF-κB and TNF-α in colonic mucosa [119].

Vanillic acid (VA) is a well-known flavonoid, which possesses various pharmacological activities such as anti-colitis, anti-mutagenic, anti-angiogenetic, anti-sickling, and anti-analgesic effects, and there is a large amount of VA in the roots of *Angelica sinensis* (Oliv.) Diels. Studies show that VA (200 mg/kg) treatment suppresses the activation of NF-κBp65 transcription in DSS-treated colon tissues and the level of IL-6, which contributes to relieving the severity of the clinical signs, including weight loss, shortening of colon length and the DAI [120].

Paeoniflorin (PA) is the main bioactive compound of Paeony root, it can be extracted from the dried peeled root of *Paeonia lactiflora* Pall. In recent study, oral administration of PA (15, 30 and 45 mg/kg) exhibits the protective effect on TNBS-induced UC mice by inhibiting MAPK/NF-κB pathway and suppressing the expressions of IL-2, IL-6, IL-10, IL-12, IL-1β, TNF-α and IFN-γ, showing PA is a novel therapeutic agent in the treatment of UC [121].

The fruit of *Ziziphus jujuba* Mill. (Rhamnaceae) has been widely used as food and Chinese medicines for over 3000 years, and described as one of the five most valuable fruits in China. Research reveals that dietary *Z. jujuba* (ZJ) fruit (5% or 10% w/w) treatment decreases fecal blood, diarrhea and DAI, and attenuates the expression of proteins in the NF-κB/IL-6/JAK1/STAT3 signaling pathway involved in inflammation associated with colorectal cancer in mice, suggesting anti-inflammatory property of ZJ [122].

Pretreatment of oligonol (10, 50 and 100 mg/kg) extracted from fruit lychee is reported to notably ameliorate pathological changes and decrease the levels of IL-1, IL-6, and TNF-α as well as NF-κB in affected colon tissues of DSS-induced UC model [123].

It is reported that *Lentinus edodes* β-glucans (500 and 1000 mg/kg) treatment can increase the body weight, improve DAI and modify p38MAPK and ERK1/2 in DSS-induced UC mice, and then phosphorylate PPARγ, which negatively regulates activation of NF-κB [124].

Study has showed that mango (*Mangifera Indica* L.) extract (10 mg GAE/L) treatment is against DSS-induced colonic inflammation and decrease NF-κB and iNOS mRNA levels, as well as NF-κB and p-NF-κB protein levels in rats. In addition, mango polyphenolics treatment attenuates the levels of inflammatory markers via suppression of the PI3K/AKT/mTOR signaling pathway, in part through up-regulation of miRNA-126 in DSS-induced colonic inflammation [125].

### Astringent medicine

Experiment shows that the activity of NF-κBp65 decreases in the colon tissue of UC female rats induced by intracolonic injection of iodoacetamide after rectal administration of muscovite treatment (360 and 720 mg/kg), which contributes to the reduction of IL-8 and TNF-α concentrations, as well as the improvements of body weight, macroscopic damage and microscopic score [126].

Shan Zhu Yu (SZY) is a dry mature flesh of the *Cornus officinalis* Sieb. et Zucc. Studies evidence that SZY (1, 10, 100 μM or 0–50 mg/mL) treatment attenuates TNF-α-induced NF-κBp65 translocation and LPS-stimulated phosphorylation and degradation of IκBα and the subsequent translocation of the p65 subunit of NF-κB to the nucleus in human umbilical vein endothelial cells and RAW 264.7 macrophage cells, respectively [127, 128]. Moreover, the isolated ursolic acid (10 and 20 mg/kg) treatment in the ethanol extract of *C. officinalis* seed inhibits phosphorylation of inhibitor of NF-κB kinase subunit β (IKKβ) and IκBα and activation of NF-κB and MAPKs in LPS-stimulated macrophages, as well as suppresses LPS-stimulated IL-1β, IL-6, TNF-α levels. Oral administration of ursolic acid significantly inhibits TNBS-induced colon shortening, and suppresses NF-κB activation in colon tissues and IL-1β, IL-6, TNF-α levels, but enhances IL-10 levels [129].

The main active compound of *Rubus coreanus* is 19a-hydroxyursane saponin. The *R. coreanus* triterpenoid compound is separated and purified from *R. coreanus* by various columns, and the structure is identified according to the physical and chemical properties and spectral data. It is found that the administration of triterpenoid component (TFRC) (25, 50 or 100 mg/kg) in *R. coreanus* improves the pathological characteristics including colon shortening and colonic epithelium injury, and reduces the expression of pro-inflammatory factors IL-1β, IL-6 and TNF-a protein and mRNA and macrophages infiltration into colon tissue in DSS-induced mice of acute UC, whose mechanisms might be related to down-regulations of NF-κB and p38MAPK signaling [130]. Furthermore, *R. coreanus* extracts (ethanol and water extracts) (400 μg/mL) treatment obviously reduces NF-κB activation and JNK and p38 phosphorylation in LPS-induced RAW 264.7 cells [131].

Osthole is an active compound isolated from the fruit of *Cnidium monnieri* (L.) Cuss. Experiment data reveals that DSS triggers the degradation of IκBα and increases the protein expression of NF-κBp65 and p-IκBα in UC mice, but after treatment with osthole (10, 20, 40 mg/kg), the expressions of NF-κBp65 and p-IκBα decrease, and expression of IκBα increases. Therefore, osthole treatment can relieve the symptoms of UC by inhibiting weight loss, colon shortening, and the DAI score, and blocking the up-regulation of TNF-α in serum and colon tissues by inhibiting the activation of NF-κB pathway [132].

## Chinese Medicine formulae targeting NF-κB pathway for UC treatment

The formulae consists of multi-Chinese medicinals and exerts synergistic effects according to the Chinese medicine therapeutic pillars based on syndrome differentiation. By focusing on the traditional functions of the formulae targeting NF-κB pathway for UC treatment, we categorized these formulas into five types.

### Tonifying Qi and activating blood category

Shen Ling Bai Zhu San (SLBZS) is one of the most popular formulae of Chinese medicine for the treatment of UC. It is composed of 10 herbs, and has been proven to have wide pharmacological effects on digestive diseases, including anti-inflammatory and gut microbiota modulation actions. In recent study, researchers apply the systems pharmacology method to explore the pharmacological mechanisms of SLBZS, and found the changes of IL-1β, IL-10, and TNF-α after SLBZS treatment (0.6 g/mL). IL-1β and TNF-α participated in the MAPK and NF-κB pathways, and the two pathways share crosstalk in DSS-induced UC rats [133].

Tou Nong San (TNS), a Chinese medicinal decoction used for treating sores and carbuncles, has a positive effect on the inflammation. TNS (3.3, 6.6 and 13.2 mL/kg) treatment leads to improvements in weight loss and water and food intake in rats. Moreover, the macroscopic and microscopic scores of rat tissues greatly decrease. Apart from that, protein and mRNA levels of proinflammatory cytokines, IL-17, TNF-α, IL-1β, and IL-6, involved in the NF-κB signaling pathway notably decrease in TNBS-induced IBD [134].

### Heat-clearing and drying dampness category

Shaoyao decoction is a Chinese medicinal formula consisted of 9 herbs. Study shows that the dose of 7.12 g/kg increases the survival rate of DSS-induced UC mice and attenuates the expression levels of serum IL-1β, IL-6, TNF-α and NF-κB activation [135].

It suggests that Qingchang Huashi granule (QCHS) (2.8, 5.5 and 11.0 g/kg) treatment inhibits the damage of colon length and ameliorates the inflammatory response by decreasing concentrations of the cytokines IL-1α, IL-6, IL-8, IL-1β, and TNF-α and increasing the concentrations of IL-4, IL-10, and IL-13 in TNBS-induced rat UC model [136]. In vitro, in TNF-α and LPS induced HT-29 cells, Qingchang Huashi Recipe (100 ng/mL, 1 and 10 μg/mL) treatment reduces the activation of NF-κB, lows the expressions of TLR4 protein and the secretion of IL-8 [137].

Study shows that administration of effective compounds alignment of Gegenqinlian decoction (GQD) (0.12 g/kg) is able to improve TNBS-induced colonic injury, which is related to significant reduction of TNF-α and IL-1β levels in colon and serum, as well as inhibiting the activation and translocation of NF-κBp65 in colon [138]. Additionally, it indicates that oral administration of GQD (0.3, 1.5 or 7.5 g/kg) alleviates the severity of colitis in DSS-induced UC mice model, and reduces TLR4 expression and NF-κB activation in mucosa, which accompanies with down-regulations of TNF-α, IL-6, IL-1β and IL-4 in the colon [139].

Jianpi Qingchang decoction (JPQCD) is a Chinese medical prescription that consists of nine Chinese herbs, namely, *Astragalus*, *Codonopsis pilosula*, *Portulaca oleracea*, *Sanguisorba officinalis*, *Notoginseng*, *Bletilla striata*, Radix Aucklandiae, and Licorice. It is reported that JPQCD (17.1 g/kg) treatment reduces the mRNA levels of IL-1β, IL-8, TNF-α and NF-κB in DSS-induced UC mice, suppresses activation of NF-κB and increases phosphorylation of IκB [140]. Using systems pharmacology to predict the active ingredients, it shows that 170 targets for the 107 active ingredients of JPQC and 112 candidate targets of UC, and JPQC treatment can improve the mucosal inflammatory response and intestinal epithelial barrier function via the NF-κB/HIF-1α signaling pathway [141].

Active compounds from modified pulsatilla decoction (MPD) have shown hepatic protective, anti‑inflammatory, antibacterial, antitumor and anti‑oxidant effects. In addition, MPD (10 mg/g body weight) treatment attenuates the severity of colitis, and suppresses the activation of the NF-κB signaling pathway in OXZ‑induced colitis mice model, and reduces the secretion of pro‑inflammatory cytokines and restores alterations in tight junction proteins in the colon tissues, showing MPD offers an effective therapeutic approach for the treatment of UC [142].

Qing Hua Chang Yin (QHCY) has been used for many years to treat conditions associated with IBD, such as UC. Moreover, QHCY (1.4 mg/mL) treatment profoundly ameliorates DSS-induced manifestations, colon shortening and histological damage in colitis mice. Moreover, its application significantly inhibits the DSS‑induced expression of TLR4 and MyD88, the phosphorylation of IκB and the nuclear translocation of NF‑κB. Taken together, it suggests that the suppression of the TLR4/NF‑κB signaling pathway may be one of the mechanisms involved in the therapeutic effects of QHCY against UC [143]. Furthermore, QHCY (1.4 mg/mL) treatment attenuates the LPS-induced phosphorylation of JAK1 and JAK2 in differentiated Caco-2 cells, which is in accordance with the reduced phosphorylation state of STAT3. It indicates that QHCY treatment inhibits the phosphorylation of STAT3, possibly through the inactivation of JAK1 and JAK2 in LPS‑induced inflammatory responses [144].

QingBai decoction (QBD) has been applied for the treatment of IBD patients in clinic. Research indicates that QBD (0.0195 mL/g) treatment effectively alleviates intestinal inflammation and mucosal barrier function in colitis mice, and reduces the production and secretion of serials pro-inflammatory cytokines such as L-1β, IL-6, and TNF-α in DSS-induced colitis. Moreover, QBD treatment decreases the intestinal permeability and inflammatory cascade by inhibiting NF-κB signaling [145].

Oral administration of Chang-An-Shuan (CAS, a 6-herb Chinese medicinal formula) at 0.5 or 5 g/kg/day ameliorates the severity of TNBS-induced colitis as evidenced by the reduced loss of body weight, alleviated diarrhea and decreased bloody stool in rats. More important, the ameliorative effects of CAS are related to the inhibition of NF-κB signaling pathway by down-regulating the expression levels of NF-κBp65, p-38 and p-AKT [146].

Baishaoqiwu (BSQW) is a formula consisting of 7 Chinese herbal medicines. Study shows that BSQW (13.2 mg/kg) treatment ameliorates TNBS-induced macroscopic and histological damage in the rats with induced colitis, and distinctly inhibits TNBS-induced expression of TLR4, MyD88 and NF-ĸBp65 genes. No treatment-related toxicity is found in the BSQW-treated group [147].

Recent results indicate that oral treatment with xie-xin decoction (2 or 4 g/kg) for 8 days promotes the recovery of colitis and inhibits the inflammatory response, including amelioration of macroscopic and histological examination, the enhanced level of IL-10, and the decreased expressions of TNF-α and NF-κBp65 in rats with experimental UC [148].

Compound sophorae decoction (CSD), consisted of 6 Chinese herbal medicines, is effective for the clinical treatment of UC. The aqueous extract of CSD (7.28 g/kg) treatment is able to improve the symptoms and pathological damage of DSS-induced UC mice, which is related to reducing the levels of IL-1β, TNF-α and phospho-NF-κBp65 and regulating Th17/Treg cell balance [149].

Huangkui Lianchang decoction (HLD) is a Chinese medicinal cocktail used to treat UC. Study indicates that HLD (9.425, 18.85, 37.70 g/kg) treatment alleviates colonic pathological damage in DSS-induced UC mice, and decreases IL-6, TNF-α, and IL-1β levels via the inhibition of the NF-κB pathway [150].

Study shows that the expressions of TLR4 and NF-κBp65 in colon tissues of UC patients obviously decrease in Bawei Xilei Powder (BXP) group compared with control group treating with hydrocortisone enema solution. BXP enema treatment (1 g/60 mL) is effective and safely applied in patients with mild to moderate UC, and its mechanisms might be involved in suppressing inflammatory response and enhancing mucosa barrier functions [151].

### Harmonizing cold and hot category

Ban-xia-xie-xin decoction (BXD) (8.7 g/kg) treatment against DSS-induced chronic UC injury in mice is characterized by amelioration of body weight loss, DAI and histology score, as well as decreasing the levels of TNF-α, IL-1β, IL-17, IL-23, COX-2 and p-p65 and increasing the level of IL-10. The protective mechanism of BXD may associate with inhibition of NF-κBp65 activation and enhancement of Nrf2 expression in colorectums of mice [152].

It is reported that wu-mei-wan (WMW) (0.515 g/mL) treatment up-regulates the expression of IL-10, down-regulates the expressions of TNF-α, IL-6, IL-8, and inhibits the NF-κBp65 activity to adjust immune function, showing WMW has better therapeutic effects on UC in rats [153].

### Warming yang and drying dampness category

Study shows that modified ZenWu decoction (MZWD) (17.47 g/kg) treatment notably reduces diarrhea, bloody stool and colon shortening and improves mucosal integrity, and suppresses inflammatory responses namely inhibiting immune-cell infiltration and serum levels of pro-inflammatory cytokines in DSS-induced experimental colitis mice. Furthermore, MZWD treatment attenuates the activation of NF-κB via lessening the degradation of IκBα in colonic tissues [154].

Ping weisan (2, 4, 8 g/kg) treatment is reported to decrease DSS-induced DAI, colon length shortening and colonic pathological damage, and reduce TNF-α, IL-1β and IL-12 secretions and suppress NF-κB pathway activation by regulating the expressions of TLR4 and PPARγ in DSS-induced chronic colitis in mice, indicating the formula might be a novel agent for the treatment of chronic colitis [155].

### Astringent category

Recent results indicate that oral treatment with zhenrenyangzang decoction (2, 4 or 8 g/kg) notably promotes the recovery of colitis which embodies the improvement of DAI and tissue damage scores in TNBS-induced UC rats, and inhibits the inflammatory response by reducing the mRNA or protein expressions of NF-κB and p38MAPK, as well as the production of TLR2 in colon tissues. The study provides direct pharmacological evidence for zhenrenyangzang decoction clinical application [156].

## Direct and indirect regulatory efficacies of Chinese medicines targeting NF-κB for UC treatment

Numerous literature have demonstrated that Chinese medicines targeting NF-κB play a vital role in UC treatment. In present study, it suggests Chinese medicines with different traditional functions exert direct and indirect regulatory efficacies on NF-κB pathway in different pathological models of UC. The definitions of direct and indirect regulatory efficacies respectively refer to action on NF-κBp65 and influence on NF-κB pathway via NF-κBp65′s up-stream or other signaling pathways.

### Direct inhibition of Chinese medicines targeting NF-κB

According to Chinese medicinal traditional functions, we summarize Chinese medicines that directly inhibit the activation of NF-κB characterized by decreasing NF-κBp65 levels, reducing the nuclear translocation of NF-κBp65 or up-regulating NF-κBp65 in cytoplasm in Table 1.

**Table 1:** Direct inhibited efficacy of Chinese medicines on NF-κB pathway

Classifications	Chinese medicines	Models	Mechanisms	References
Heat-clearing and dampness-drying medicine	Casticin	RAW264.7	Repress the NF-κBp65 nucleus translocation	[44]
	Baicalin	DSS-induced colon tissue	Reduce the protein levels of NF-κBp65 and p-NF-κBp65	[45]
		UC patients	Decrease ratios of p-NF-κB/NF-κB	[46]
	Wogonin	Caco-2 cells	Decrease NF-κB activity	[47]
	Wogonoside	DSS-induced colon tissues	Inhibit nuclear translocation of NF-κBp65, phosphorylated p65 and NF-κB DNA binding activity	[48]
		AOM/DSS-induced tumor tissues	Inhibit the protein expression of p-p65	[49]
		THP-1 cells	Suppress NF-κB nuclear translocation	[49]
	Berberine	Colonic macrophages and epithelial cells	Inhibit activation of NF-κB	[50]
	(±)-8-ADC	DSS-induced colon tissues	Reduce phosphor-NF-κBp65 expression; Decrease the NF-κB mRNA expression	[53]
		IEC6 Cells	Reduce phosphor-NF-κBp65 expression	[53]
	Berberine hydrochloride	DSS-induced colonic tissues	Suppress the expressions of phosphorylation of NF-κBp65	[54, 55]
	Apocynin	DSS-induced tissues	Reduce the activation of NF-κBp65	[56]
	Matrine	TNBS-induced colonic mucosa	Reduce the overexpression of NF-κBp65	[58]
	Oxymatrine	TNBS-induced colon mucosa	Down-regulate the expression of NF-κBp65	[59]
	Ampelopsis grossedentata	DSS-induced colon tissues	Suppress the NF-κB activation	[60]
	SM934	DSS-induced colon tissue	Decrease phosphorylation of NF-κB	[61]
		Macrophages	Suppress phosphorylation of NF-κBp65; Inhibit NF-κB nuclear translocation	[61]
	Artesunate	RAW264.7 cells	Reduce the expression levels of p‑NF‑κB	[62]
	Tetrandrine	DSS-induced colon tissue	Inhibit the binding activity of NF-κB to DNA	[63]
	*Polygonum cuspidatum* Siebold & Zucc.	DSS-induced colon tissue	Suppress the NF-κB	[64]
Interior-warming and drying dampness medicine	Aesculin	RAW264.7 cells	Inhibit p-NF‑κBp65 levels in nucleus	[65]
	Total triterpenoids of *Chaenomeles speciosa*	DSS-induced colon tissue	Down-regulate the protein expressions of nucleus NF-κBp65, up-regulate the protein expressions of cytosol NF-κBp65	[66]
	Cardamonin	RAW264.7 cells	Prevent the nuclear accumulation of NF‑κBp65; Block the translocation of NF‑κBp65	[67]
		AA-induced colon tissue	Reduce the levels of NF-κB	[69]
	3,4-Oxo-isopropylidene-shikimic acid	TNBS-induced colon tissues	Decrease NF-κBp65 subunit level	[70]
	Wu Zhu Yu	DSS-induced colon tissues	Down-regulate the increased phosphorylation levels of NF-κBp65	[71]
	Clove	TNBS-induced colon tissue	Down-regulate the expressions of NF-κBp65	[72]
		DSS-induced colon tissues	Reduce the expressions of p65-NF-κB	[73]
		RAW264.7 macrophages	Decrease the expressions of p65-NF-κB	[73]
	*Zanthoxylum bungeanum*	DSS-induced colon tissue	Inhibit NF-κBp65 phosphorylation levels	[74]
	Galangin	DSS-induced colon tissue	Suppress protein expressions of p-NF-κB; Decrease the accumulation of nuclear NF-κBp65	[75]
Detoxificating and purgative medicine	Parthenolide	DSS-induced colon tissue	Inhibit the phosphorylation of NF-κBp65	[76]
		HCT116 cells	Block the activation of p65	[77]
	Arctigenin	DSS-induced colonic tissues	Suppress phosphorylation of NF-κBp65 and p65 translocation	[78]
	Andrographolide derivative AL-1	TNBS-induced colon tissues	Aattenuate the expression levels of p-p65	[79]
	Andrographolide derivative CX-10	DSS-induced colonic tissue	Reduce the expressions of NF-κB p65	[80]
	Brusatol	DSS-induced colon tissue	Attenuate the expression of NF-κBp65	[81]
	*Brucea javanica* oil	DSS-induced colon tissues	Suppress the NF-κB activation; Inhibit the phosphorylation of NF-κBp65	[82]
	Total flavonoids of *Hedyotis diffusa* Willd	RAW 264.7 cells	Reduce the phosphorylation of NF-κBp65	[83]
	*Oldenlandia diffusa*	DSS-induced colon tissues	Reduce the activation of NF-κBp65	[84]
	Qing Dai	RAW264.7 cells	Abolish NF‑κBp65 translocation to the nucleus	[86]
	Indirubin	DSS-induced colon tissues	Reverse DSS-mediated up-regulation of p-NF-κBp65	[87]
	Dandelion	DSS-induced NCM460 cell	Decrease the phosphorylation of NF‑κBp65	[88]
	*Portulaca oleracea* L.	DSS-induced colon tissues	Reduce the protein expressions of NF-κBp65	[89]
	Rhubarb-type anthraquinones	RAW264.7 macrophages	Rhein reduce phosphor-NF-κBp65 levels	[91]
		HT-29 cells	Emodin block NF-κBp65 nuclear translocation	[92]
		DSS-induced colitis tissues	Chrysophanol reduce the activation of NF-κBp65	[93]
	Gallic acid	DSS-induced colon tissue	Inhibit the nuclear translocation of NF-κBp65	[94]
		RAW264.7 macrophage	Decrease the expression of NF-κBp65	[94]
	Allicin	DSS-induced colonic mucosa	Prevent the inhibition of inducing nuclear translocation of NF-κBp65	[95]
	*Cassia obtusifolia*	DSS-induced colon tissues	Reduce the level of NF-κBp65	[96]
	Bergenin	RAW264.7 cells	Suppress the nuclear translocation and DNA-binding activity of NF-κBp65; Inhibit the acetylation of NF-κBp65, increase the association of NF-κBp65 and IκBα, and hinder the nuclear translocation of NF-κBp65	[97]
	*Fagopyrum cymosum* (Trev.) Meisn	Raw264.7 cells	Inhibit NF-κBp65 nuclear translocation	[98]
	Chlorogenic acid	DSS-induced colon mucosa	Reduce the phosphorylation level of NF-κBp65 protein	[99]
Blood-activating medicine	Paeonol	CW-2 cells	Reduce NF-κB transactivation	[99]
	Liriodendrin	DSS-induced colon tissues	Suppress the activation of NF-κB pathways; Reduce in phosphorylation of NF-κB	[101]
		RAW264.7 macrophages	Reduce the phosphorylation of NF-κB	[101]
	Shikonin	DSS-induced colon tissues	Reduce the expression of NF-κBp65	[102]
	Tetramethylpyrazine	OXZ-induced colitis mucosa	Reduce the rise in NF-κBp65	[103]
	Crocetin	TNBS-induced colonic mucosa	Down-regulate the NF-κB	[104]
	Epicatechin	RAW264.7 cells	Inhibit the activation of NF-κB	[105]
	Curcumin	Colonic mucosa	Modulate NF-κB activation	[110]
Qi-regulating medicine	Norisoboldine	DSS-induced colon tissues	Inhibit phosphorylation, nuclear translocation and DNA-binding activity of NF-κBp65	[113]
	*Citrus aurantium* L.	TNBS-induced colon tissues	Inhibit the NF-κB pathway	[114]
		RAW264.7 cells	Inhibit the protein expressions of NF-κB	[114]
Tonifying medicine	*Eclipta Prostrata*	HT-29 cells	Reduce the nuclear translocation of NF-κB	[115]
	Astragalus polysaccharide	DSS-induced colonic tissues	Reduce NF-κB DNA phosphorylation activity	[116]
	Astragaloside IV	CCD‑18Co cells	Inhibit the phosphorylation of NF‑κBp65	[117]
	Licochalcone A	DSS-induced colonic tissues	Reverse the increased expression of p65 NF-κB	[118]
	Diammonium glycyrrhizinate	AA-induced colonic tissues	Suppress the positive percentage and density of NF-κBp65	[119]
	Vanillic acid	DSS-induced colon tissues	Suppress the activation of transcription NF-κBp65	[120]
	Paeoniflorin	TNBS-induced colon tissues	Inhibit the expressions of p-NF-κB	[121]
	The fruit of *Ziziphus jujuba* Mill.	AOM/DSS-induced colon tissue	Attenuate the expression of proteins in the NF-κB	[122]
	Oligonol	DSS-induced colon tissue	Decrease nuclear translocation of NF-κBp65	[123]
	*Lentinus edodes* β-glucans	RAW264.7 cells	Inhibit NF-κB activation	[124]
	Mango extract	CCD-18Co cells	Reduce expression of NF-κB and pNF-κB protein	[125]
Astringent medicine	Muscovite	Iodoacetamide-induced colitis tissues	Decrease the activity of NF-κBp65 and reduce the p65 transferred into the nucleus	[126]
	Shan Zhu Yu	Human umbilical vein endothelial cells (HUVECs)	Attenuate NF-κB expression	[127]
		Macrophages	Inhibit NF-κB activation	[129]
	*Rubus coreanus*	RAW264.7 macrophages	Attenuate the nuclear translocations of NF-κBp65	[130]
		RAW 264.7 cells	Reduce NF-κB activity	[131]
	Osthole	DSS-induced colonic tissue	Reduce the expression of NF-κBp65	[132]
Tonifying Qi and activating blood category	Tou Nong San	TNBS-induced colon tissue	Reduce the activation of the NF-κBp65	[134]
Heat-clearing and drying dampness category	Shaoyao Decoction	AOM/DSS-induced colonic tissues	Suppress NF-κB activation	[135]
	Qingchang Huashi recipe	HT-29 cells	Reduce the activation of NF-κB	[137]
	Gegenqinlian Decoction	TNBS-induced colon tissue	Inhibit the activation and translocation of NF-κBp65	[138]
		DSS-induced colonic tissues	Decrease the P-NF-κBp65	[139]
		RAW 264.7 cells	Down-regulate the expression of P-NF-κBp65	[139]
	Jianpi Qingchang decoction	DSS‑induced colon tissue	Inhibit the activation of the NF-κB	[140]
	Modified pulsatilla decoction	OXZ-induced colon tissues	Suppress the activation of the NF-κB	[142]
	Qing Hua Chang Yin	DSS‑induced colon tissue	Inhibit the expression of the nuclear translocation of NF‑κB	[143]
	QingBai decoction	DSS‑induced colon tissue	Decrease the protein level of P-NF-κBp65	[145]
	Chang-An-Shuan	TNBS-induced colonic tissues	Down-regulate the expression levels of NF-κBp65	[146]
	Baishaoqiwu	TNBS-induced colon tissues	Inhibit expression of the NF-ĸBp65 genes	[147]
	Xie-xin decoction	TNBS-induced colon tissues	Decrease expression of NF-ĸBp65	[148]
	Compound sophorae decoction	DSS‑induced colonic tissues	Reduce the level of phospho-NF-κBp65	[149]
	Huangkui Lianchang decoction	DSS-induced Colon Tissue	Decrease the NF-B levels	[150]
	Bawei Xilei powder	UC patients	Decrease the expression of NF-κBp65	[151]
Harmonizing cold and hot category	Ban-xia-xie-xin decoction	DSS-induced colorectums	Inhibition of NF-κBp65 activation	[152]
	Wu-mei-wan	DNCB-induced colonic tissue	Inhibit the NF-κBp65 activity	[153]
Warming yang and drying dampness category	Modified ZenWu decoction	DSS-induced colonic tissues	Attenuate the activation of NF-κB and suppress the expression of NF-κBp65	[154]
	Ping weisan	DSS-induced colon tissues	Reduce phosphorylation of NF-κBp65	[155]
		RAW264.7cells	Reduce phosphorylation of NF-κBp65	[155]
Astringent category	Zhenrenyangzang decoction	TNBS-induced colon tissues	Reduce NF-κB mRNA expression	[156]

### Indirect regulation of Chinese medicines targeting NF-κB

Apart from direct inhibited effect, Chinese medicines can indirectly regulate NF-κB pathway as well. For example, Chinese medicines increase IκBα levels, inhibit IκB protein phosphorylation or modulate the association between NF-κBp65 and IκBα, as well as act on other signaling pathways to influence the activation of NF-κB pathway in pathogenic mechanisms of UC [157]. Based on Chinese medicinal traditional functions, we summarize Chinese medicines indirectly regulate NF-κB pathway in Table 2.

**Table 2:** Indirect regulatory efficacy of Chinese medicines on NF-κB pathway

Classifications	Chinese medicines	Models	Mechanisms	References
Heat-clearing and dampness-drying medicine	Casticin	RAW264.7	Down-regulate the phosphorylation of AKT, thus down-regulate the phosphate kinase activity of IKKα/β, thereby failing to phosphorylate IκBα	[44]
	Baicalin	DSS-induced colon tissue	Reduce the protein levels of p-IκB-α, whereas IκB-α protein expression is increased	[45]
		UC patients	Increase p-STAT6/STAT6 ratio, but decrease ratios of p-STAT4/STAT4	[46]
	Wogonin	Caco-2 cells	Decrease phosphorylation and degradation of IκB; inhibit the expression of TLR4, MyD88 and TAK1	[47]
	Wogonoside	DSS-induced colon tissues	Inhibit phosphorylated IκBα	[48]
		AOM/DSS-induced tumor tissues	Decrease the phosphorylation of IKKα and IκBα, inhibit NF-κB activation via PI3K/Akt pathway	[49]
		THP-1 cells	Inhibit phosphorylation of IKKα and IκBα	[49]
	Berberine	Colonic macrophages	Inhibit ERK1/2, p38, and JNK activation; inhibit IκB degradation	[50]
		Colonic epithelial cells	Decrease IκB degradation and ERK1/2 and p38 activation	[50]
	Berberine hydrochloride	DSS-induced colonic tissues	Suppress the expressions of phosphorylation of STAT3; inhibit the activation of IL-6/STAT3/NF-κB	[54, 55]
	Apocynin	DSS-induced tissues	Reduce the activation of STAT3	[56]
	SM934	DSS-induced colon tissue	Decrease phosphorylation of IκB; abrogate the increased phosphorylation of ERK1/2 induced by DSS	[61]
Interior-warming and drying dampness medicine	Aesculin	RAW264.7 cells	Suppress the phosphorylation of IκBα	[65]
	Total triterpenoids of *Chaenomeles speciosa*	DSS-induced colon tissue	Down-regulate the protein expressions of cytosol PPARγ, tissue p-IKKβ and p-IκBα, decrease the p-IKKβ/IKKβ and p-IκBα/IκBα ratios, up-regulate the protein expressions of nucleus PPARγ, tissue SIRT1	[66]
	Cardamonin	RAW264.7 cells	Inhibit phosphorylation and degradation of IκBα; inhibit p38 phosphorylation	[67]
	3,4-Oxo-isopropylidene-shikimic acid	TNBS-induced colon tissues	Decrease the change of IκBα expression in the nucleus	[70]
	Wu Zhu Yu	DSS-induced colonic tissues	Down-regulated the increased phosphorylation levels of IκB	[71]
	Clove	DSS-induced colon tissues	Reduce the expressions of p-IκB-α	[73]
		RAW264.7 macrophages	Decrease the expressions of p-IκB-α	[73]
	*Zanthoxylum bungeanum*	DSS-induced colon tissue	Inhibit IκBα phosphorylation levels; suppress NF-κB due to inhibition of TLR4	[74]
	Galangin	DSS-induced colon tissue	Suppress protein expressions of p-Ikk-βα; increase the expression level of phosphorylation of IκBα in the cytoplasmic fraction	[75]
Detoxificating and purgative medicine	Parthenolide	DSS-induced colon tissue	Inhibit IkB protein phosphorylation; block the phosphorylation and degradation of IκBα	[76]
		HCT116 cells	Inhibit the phosphorylation of IkB-α	[77]
	Arctigenin	DSS-induced colonic tissues	Suppress phosphorylation of IκBα	[78]
	Andrographolide derivative AL-1	TNBS-induced colon tissues	Aattenuate the expression levels of p-IκBα; increase the expression of PPAR-γ	[79]
	Andrographolide derivative CX-10	DSS-induced colonic tissue	Reduce the expressions of p-IκBα; increase the expression of IκBα and suppresses the phosphorylated p38MAPK, ERK and JNK	[80]
	*Brucea javanica* oil	DSS-induced colon tissues	Inhibit the phosphorylation of IκBα	[82]
	Total flavonoids of *Hedyotis diffusa* Willd	RAW 264.7 cells	Suppress IκB phosphorylation, and reduce the phosphorylation of MAPK	[83]
	Qing Dai	RAW264.7 cells	Inhibit IkBα degradation	[86]
	Indirubin	DSS-induced colon tissues	Reverse DSS-mediated up-regulation of p-IκBα and p-IKKα/β as well as down-regulation of IκBα and IKKα/β	[87]
	Dandelion	DSS-induced NCM460 cell	Decrease the phosphorylation of Akt	[88]
	Rhubarb-type anthraquinones	HT-29 cells	Emodin up-regulating the expression of IκB	[92]
		Macrophages	Chrysophanol inhibit IκBα degradation	[93]
	Gallic acid	DSS-induced colon tissue	Attenuate the activation and nuclear accumulation of p-STAT3 (Y705); prevent the degradation of the inhibitory protein IκB	[94]
	Allicin	DSS-induced colonic mucosa	Inhibit activation and nuclear accumulation of STAT3; prevent the inhibition of protein IκB degradation	[95]
	Bergenin	RAW264.7 cells	Activate PPARγ, leading to increased expression of SIRT1	[97]
	*Fagopyrum cymosum* (Trev.) Meisn	RAW264.7 cells	Inhibit IκB phosphorylation	[98]
	Chlorogenic acid	DSS-induced Colon Mucosa	Reduce the phosphorylation level of IκB; improve the expression of ERK1/2, p-ERK, p38, p-p38, JNK, and pJNK proteins of the MAPK/ERK/JNK pathway	[99]
Blood-activating medicine	Liriodendrin	DSS-induced colon tissues	Reduce in phosphorylation of Akt; Block the phosphorylation of IκBα	[101]
		RAW264.7 macrophages	Reduce the phosphorylation of IκBα	[101]
	Shikonin	DSS-induced colon tissues	Reduce the expression of pSTAT-3	[102]
	Tetramethylpyrazine	OXZ-induced colitis mucosa	Restore PPAR-γ expression; reverse increased p38 MAPK phosphorylation	[103]
	(2)-epigallocatechin-3-gallate	IEC-6 cells	Inhibit the activation of IκB kinase	[106]
	Curcumin	DSS-induced colonic tissue	Inhibit the activating signals of p38MAPK	[112]
Qi-regulating medicine	Norisoboldine	DSS-induced colon tissues	Repress the phosphorylations of p38MAPK and ERK	[113]
Tonifying medicine	*Eclipta Prostrata*	HT-29 cells	Reduce the degradation of IκB	[115]
	Astragaloside IV	CCD‑18Co cells	Inhibit the phosphorylation of IκB	[117]
	Licochalcone A	DSS-induced colonic tissues	Reverse the increased expression of IKKα and p-IκB	[118]
	The fruit of *Ziziphus jujuba* Mill.	AOM/DSS-induced colon tissue	Attenuate inflammation, tumor development and progression by down-regulation of expression of STAT3	[122]
	*Lentinus edodes* β-glucans	DSS-induced colon tissues	Modify p38MAPK and ERK1/2, and then phosphorylating PPARγ, which negatively regulates activation of NF-κB	[124]
	Mango polyphenolics	DSS-induced intestinal mucosa	Attenuate the levels of inflammatory markers via suppression of the PI3K/AKT/mTOR signaling pathway, in part through up-regulation of miRNA-126	[125]
Astringent medicine	Ursolic acid	Macrophages	Inhibit the phosphorylation of IKKβ and IκBα; suppress MAPK signaling pathways	[129]
	*Rubus coreanus*	RAW264.7 macrophages	Block the IκBα phosphorylation and degradation of IκBα; suppression of MAPK activation	[130]
	Osthole	DSS-induced colonic tissue	Reduce the expression of p-IκBα and increase IκBα expression	[132]
		RAW 264.7 cells	Reduce the phosphorylation of the MAPK/p38 protein; Facilitate a full recovery of the degradated IκBα protein	[132]
Tonifying Qi and activating blood category	Shen Ling Bai Zhu San	DSS-induced colonic tissue	MAPK and NF-κB pathways share crosstalk	[133]
	Tou Nong San	TNBS-induced colon tissue	Inhibit p65 mainly by inhibiting the phosphorylation of IKK*β* and thus the degradation of IB	[134]
Heat-clearing and drying dampness category	Gegenqinlian decoction	DSS-induced colonic tissues	Decrease the P-IκB	[139]
		RAW 264.7 cells	Inhibit the degradation of IκB	[139]
	Jianpi Qingchang decoction	DSS‑induced colon tissue	Increase the expression of IκB	[140]
	Qing Hua Chang Yin	DSS‑induced colon tissue	Inhibit the expression of the phosphorylation of IκB	[143]
		Caco-2 cells	Suppress the phosphorylation of JAK1, JAK2 and STAT3	[144]
	QingBai decoction	DSS-induced colon tissue	Strengthen mucus barrier through inhibiting p‐ERK and Notch signaling	[145]
	Chang-An-Shuan	TNBS-induced colonic tissues	Reduce the levels of p38 and p-AKT proteins	[146]
	Compound sophorae decoction	DSS‑induced colonic tissues	Suppress the activation of STAT3	[149]
	Huangkui Lianchang decoction	DSS-induced Colon Tissue	Decrease the IκBα and p-IκBα levels	[150]
Warming yang and drying dampness category	Modified ZenWu decoction	DSS-induced colonic tissues	Lessen the degradation of IκBα	[154]
	Ping weisan	DSS-induced colon tissues	Reduce phosphorylation of IκBα	[155]
		RAW264.7cells	Reduce phosphorylation of IκBα	[155]
Astringent category	Zhenrenyangzang decoction	TNBS-induced colon tissues	Attenuate the protein expression of IκB-α subunit and phosphorylated p38MAPK (p-p38MAPK)	[156]

## Conclusion

In terms of direct inhibition of NF-κB in the treatment of UC, we summarized 85 kinds of Chinese medicines and their active compounds, as well as formulae. By analysis, we find that four categories of heat-clearing and dampness-drying medicine, interior-warming and drying dampness medicine, detoxificating and purgative medicine and tonifying medicine account for primary status, which embodies prevention of phosphorylated and nuclear transcriptional NF-κBp65 or enhancement of the level of p65 in the cytoplasm. Simultaneously, we also discover that the number of formulae with function of heat-clearing and drying dampness targeting direct inhibition of NF-κB is maximum.

On the other hand, we summarized 58 Chinese medicines and their active compounds, as well as formulae for indirect inhibition of NF-κB. Among them, the categories of heat-clearing and dampness-drying medicine and formulae, and detoxificating and purgative medicine exert distinguished actions, which embodies enhancing the activity of IκBα to decrease phosphorylated p65 or inhibiting the degradation of IκBα binding to p65. Apart from affecting IκBα, some Chinese medicines and their active compounds are able to regulate the activity of NF-κB by affecting AKT, TLR4/MyD88, PPARγ/SIRT1, p-IKKβ/IKKβ, p38MAPK/ERK/JNK, TLR5/MyD88, p-STAT3. As shown in Tables 1 and 2, from the analysis on the frequency of Chinese medicines with different therapeutic function for treating UC, it suggests that the screening of Chinese medicines for UC treatment might be focused on the candidate agents with dampness-drying and detoxificating effects in future.

Furthermore, cell-specific role of NF-κB has been demonstrated to involve in the pathogenesis of IBD [158]. It suggests the enhancement of NF-κBp65 exists in macrophages and epithelial cells isolated from inflamed gut specimens from IBD patients [159]. Except for macrophages and epithelial cells, lamina propria fibroblasts also plays a NF-κB mediated pro-inflammatory role in IBD [160]. Increased NF‐κB expression in mucosal macrophages causes the productions of pro-inflammatory cytokines such as TNF‐α, IL‐1 and IL‐6 directly resulting in the mucosal tissue damage, and in colonic epithelial cells it is related to an increased expression of intercellular adhesion molecule‐1 that contributes to the recruitment of neutrophil granulocytes to the site of inflammation [161]. NF‐κB‐induced cytokines further stimulate, activate and differentiate lamina propria immune cells, which aggravates the perpetuation of mucosal inflammation. In this review, we find that 55% of Chinese medicines having the abilities of regulating NF‐κB expression in macrophages (e.g., SM934, artesunate, aesculin, gallic acid and epicatechin) repress the nucleus translocation and phosphorylation of NF-κBp65, inhibit the protein expressions and phosphorylation of NF-κB, whereas 45% (e.g., berberine, Total flavonoids of *H. diffusa* Willd, *F. cymosum* (Trev.) Meisn and osthole) block the phosphorylation of IκB, reduce the degradation of IκBα protein. It suggests that Chinese medicines with detoxificating and purgative function have the most obvious inhibited effects of NF-κB pathway on macrophages. Additionally, other active compounds or extracts of Chinese medicines, such as Chinese herb pair Paeoniae Radix Alba and Atractylodis Macrocephalae Rhizoma, *Corni Fructus* aqueous extract and cornuside, *Siegesbeckia pubescens* Makino, which can inactivate NF-κB pathway in macrophage, perhaps became candidate drugs for treating UC in future [162–164].

Apart from NF-κB, Nrf2 is also a key transcription factor that protects cells and tissues from inflammation, and it has been found to be implicated in the pathogenesis of UC [165]. Study reports that when Nrf2 expression rises in colon tissue, the inflammatory injury significantly attenuates, demonstrating that Nrf2 has a protective effect on the colon [166]. Meanwhile, scholars find that there are multiple associations between Nrf2 and NF-κB [167]. For example, the absence of Nrf2 leads to oxidative stress activation, which contributes to increasing NF-κB-mediated cytokines expression. In present study, we find some active compounds of Chinese medicines and formulae, e.g., galangin, oligonol, bai-xia-xie-xin decoction, can either increase the nuclear translocation of Nrf2 or inhibit NF-κB pathway, suggesting the advantages of Chinese medicines’ multi-targets and comprehensive regulated abilities for UC treatment.

Additionally, numerous literature have reported Chinese herbal drugs administered by both, oral intake and retention enema, exert significant therapeutic actions in the treatment of IBD, and in order to understand the detailed mechanisms of Chinese medicines therapy on UC, the application of experimental rodent models is essential [168, 169]. In this review, we summarized the researches mainly from animal or human cell models, e.g. DSS-induced model is common pathological UC model. It suggests that the establishment of chronic colitis and the development of colorectal dysplasias and cancers with pathological features by DSS-induced rodent models resemble those of human colitis, suggesting that DSS-induced colitis may be an appropriate model to evaluate the clinical efficacy of candidate agents for UC [170, 171]. Therefore, we can infer some characteristics of Chinese medicine from analyzing UC animal models based on traditional Chinese herbal therapeutic functions. In addition, the development of novel deliver Chinese medicines for the treatment of IBD will more greatly enhance the therapeutic effects and safety of Chinese medicines, as well as reduce the cost of treatment [172].

**Fig. 1:**
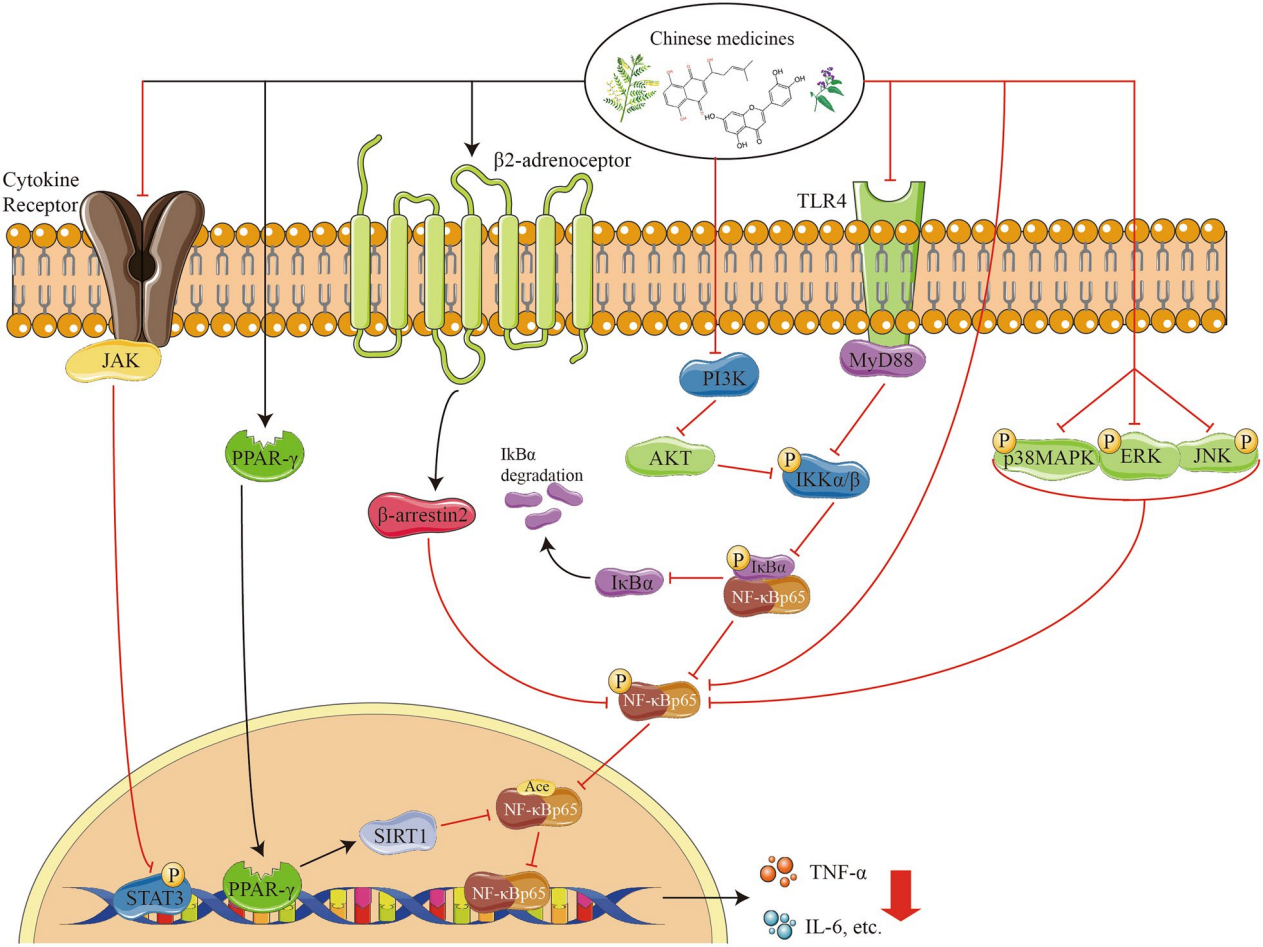
The schematic diagram of Chinese medicines direct and indirect regulating NF-κB pathway in UC treatment

In summary, evidences show that Chinese medicines exert a vital action on the treatment of UC by direct and indirect regulation of NF-κB pathway (Fig. 1), and the category of dampness-drying and detoxificating medicine accounts for the main status. This review not only further validates the scientificity of Chinese medicine on the pathogenesis of UC by categorized therapeutic agents and formulae according to their traditional functions, but also suggests the characteristics of comprehensive modulated abilities of Chinese medicines. Moreover, by careful analysis on cell-specific role of NF-κB in UC, novel Chinese medicines precisely modulate NF-κB becomes possible. Simultaneously, this review contributes to the choices of Chinese medicine category and provides curative potential of Chinese medicines for clinical UC treatment. Of course, more systematic and detailed pharmacological studies on Chinese medicines need to be fulfilled and provide more evidences to validate Chinese medicines’ efficacy and safety in the future.

## Data Availability

Not applicable.

## Abbreviations

COX2: Cyclooxygenase-2
DSS: Dextran sulfate sodium
ERK: Extracellular regulated protein kinases
IFN-γ: Interferon gamma
IKKβ: Inhibitor of nuclear factor κB kinase subunit β
IL: Interleukin
iNOS: Inducible nitric oxide synthase
JNK: c-Jun N-terminal kinase
LPS: Lipopolysaccharide
MAPK: Mitogen-activated protein kinase
MLC: Myosin light chain
MyD88: Myeloid differentiation factor 88
NO: Nitric oxide
OXZ: Oxazolone
PGE2: Prostaglandin E2
PPAR-γ: Peroxisome proliferator-activated receptor gamma
ROS: Reactive oxygen species
STAT3: Signal transducer and activator of transcription 3
TLR4: Toll like receptor 4
TNBS: Trinitrobenzene sulfonic acid
TNF: Tumor necrosis factor
UC: Ulcerative colitis
